# PhysioDirect: Supporting physiotherapists to deliver telephone assessment and advice services within the context of a randomised trial

**DOI:** 10.1016/j.physio.2012.08.002

**Published:** 2013-06

**Authors:** Annette Bishop, Jill Gamlin, Jeanette Hall, Cherida Hopper, Nadine E. Foster

**Affiliations:** a Arthritis Research UK Primary Arthritis Research UK Primary Care Centre, Primary Care Sciences, Keele University, Staffordshire, ST5 5BG, United Kingdom; b Physiotherapy Department, Hinchingbrooke Hospital, Hinchingbrooke Park, Huntingdon, Cambs PE29 6DN, United Kingdom; c Bristol Community Health, Physiotherapy department, Knowle Clinic, Broadfield Road, Knowle, Bristol, BS4 2UH, United Kingdom; d Academic Unit of Primary Health Care, School of Social and Community Medicine, University of Bristol, Canynge Hall, 39 Whatley Road, Bristol, BS8 2PS, United Kingdom

**Keywords:** Musculoskeletal, Telemedicine, Physiotherapy, Accessibility of health services

## Abstract

Physiotherapy-led telephone assessment and advice services for patients with musculoskeletal problems have been developed in many services in the UK, but high quality trial data on clinical and cost effectiveness has been lacking. In order to address this ‘The PhysioDirect trial’ (ISRCTN55666618), was a pragmatic randomised trial of a PhysioDirect telephone assessment and advice service. This paper describes the PhysioDirect system used in the trial and how physiotherapists were trained and supported to use the system and deliver the PhysioDirect service. The PhysioDirect system used in the trial was developed in Huntingdon and now serves a population of 350,000 people. When initiating or providing physiotherapy-led telephone assessment and advice services training and support for physiotherapists delivering care in this way is essential. An enhanced skill set is required for telephone assessment and advice particularly in listening and communication skills. In addition to an initial training programme, even experienced physiotherapists benefit from a period of skill consolidation to become proficient and confident in assessing patients and delivering care using the telephone. A computer-based system assists the delivery of a physiotherapy-led musculoskeletal assessment and advice service.

Clinical Trials Registration Number (ISRCTN55666618).

## Introduction

Physiotherapy-led telephone assessment and advice services have been developed in many services in the UK in an attempt to meet demand for physiotherapy services and improve patient outcomes by reducing waiting times to first physiotherapy contact [1]. Such service developments mean that physiotherapists are being asked to deliver care in new and innovative ways.

The ‘PhysioDirect trial’ (ISRCTN55666618), funded by the Medical Research Council, was a pragmatic randomised trial of a PhysioDirect telephone assessment and advice service, to compare the clinical and cost-effectiveness of PhysioDirect with usual physiotherapy care for adults with musculoskeletal problems. The design of the trial is detailed elsewhere [2].

This paper complements the protocol and main trial results papers by describing the PhysioDirect system used in the trial and how physiotherapists were prepared for using the system and delivering the service, including the training and competency assessment process.

## The PhysioDirect system

There are many telephone assessment and advice systems around the UK that can be included under the umbrella term of ‘PhysioDirect’. The PhysioDirect system used in the MRC-funded PhysioDirect trial was developed in Huntingdonshire Primary Care Trust (PCT) in 2001 and currently serves 350,000 people in Cambridgeshire. The established nature of the service, the structured format of the system, and the experience of the Huntingdon physiotherapy staff both in using and in training other physiotherapists in using this system, made the system particularly suitable for use in the PhysioDirect trial.

**Fig. S1:**
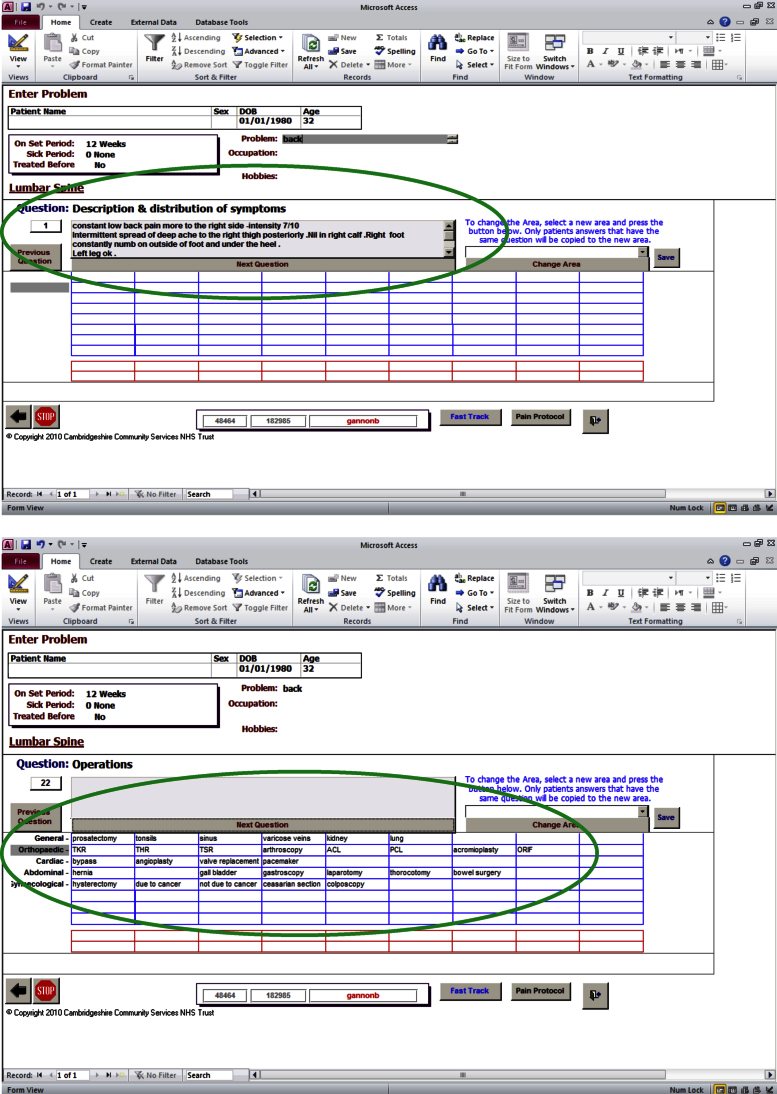
Example screen shots from the PhysioDirect system.

Shortly after its inception, the Huntingdon PhysioDirect system was translated into a computer-based system to assist the safe, efficient and effective delivery of the telephone assessment and advice service. The challenge during the development of the computerised system was to maintain sufficient structure to guide an effective and efficient assessment by prompting physiotherapists to cover all key aspects of the patient assessment, whilst offering sufficient flexibility to be responsive to the variety of presentations of individual patients. The system had to be as simple as possible as physiotherapists undertaking telephone assessment are required to perform a number of skills simultaneously, including asking questions, visualising the patient and their presentation, analysing the patient's responses, formulating the next question and typing responses into the computer-based system. The solution was to include mandatory fields with drop down menus and tick boxes for key aspects of the assessment with text boxes provided to allow the physiotherapists to record responses to supplementary questions eliciting further information or clarification from the patient. There are two example screen shots shown in Supplementary data Fig. S1, with the first showing the free text box for the description and distribution of the patient's symptoms and the second showing a drop down menu, in this example for previous surgical interventions to be recorded.

The physiotherapist selects an appropriate assessment framework, depending on the area of the body affected, which prompts questions for the physiotherapist to assess the patient and reach a clinical diagnosis. The therapist can change between algorithms should the clinical presentation of the patient indicate that other bodily areas also need to be considered. The telephone assessment results in each patient being categorised into one of five categories. The categories and the subsequent management pathways are summarised in Table 1.

The clinical algorithms then guide the physiotherapist to the most appropriate clinical management pathway. Using the ankle as an example, there are four recommended pathways:

• Referral directly to the Accident and Emergency department for suspected fractures
• A face to face appointment for those with significant swelling or difficulty in weight bearing
• An initial trial of advice and home exercises (1 to 2 weeks) for mild acute onset problems (such as mild sprains and strains) or a flare or recurrence of symptoms related to an established condition such as osteoarthritis
• A home exercise programme (∼6 weeks) for chronic musculoskeletal problems

In addition to guiding the assessment and recording the clinical diagnosis and patient management pathway, the computerised system also includes an algorithm to support physiotherapists to provide safe advice to patients on over-the-counter medication, developed by Hinchingbrooke Hospital pharmacy department. Effective pain management is crucial in the early management of musculoskeletal problems, providing an opportunity for the rehabilitation of normal movement and is a key factor in the success of self-management approaches such as many of the management pathways utilised through PhysioDirect. Currently, undergraduate physiotherapy training does not include training in medication advice and the number of physiotherapist supplementary prescribers is small, only 219 in late 2011 [3]. Given this, the pharmacy algorithm in the PhysioDirect system allows consistent and safe advice on analgesia to be provided by physiotherapists without the need for the patient to subsequently see their GP or pharmacist thus reducing delays in commencing effective self-management.

## Skills for effective telephone assessment and advice

**Fig. 1:**
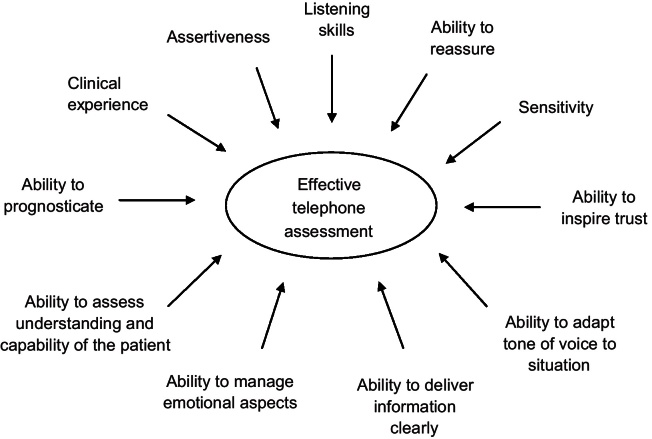
Physiotherapist skills required for effective telephone assessment.

Telephone assessment requires an enhanced skill set for the physiotherapist as communication is limited to audible information only for both the physiotherapist and patient. The Huntingdon service's experience is that physiotherapists conducting telephone assessment must be clinically experienced in the diagnosis of musculoskeletal conditions and usual physiotherapy service pathways and be able to provide information in a clear and unambiguous manner that individual patients understand. In addition, listening skills are one of the most important aspects of assessing patients by telephone [4–6]. Despite the lack of visual cues, factors such as the tone of voice of the patient, signs of emotions such as tears or sighing, or marked silences can provide valuable insights into how the patient is dealing with their condition [4–6]. The skill set that the Huntingdon team suggest may need to be enhanced to deliver effective telephone assessment and advice are shown in Fig. 1.

## Training physiotherapists to use the PhysioDirect system

Training in the delivery of telephone assessment and advice is an essential part of initiating and providing a PhysioDirect service. Even experienced musculoskeletal physiotherapists need to be trained in the new system, supported to develop the enhanced skill set and given the opportunity to consolidate their skills so that they feel confident and competent in delivering care in this way.

In preparing Huntingdon's own staff for working in the PhysioDirect service, an individual's clinical reasoning and experience is assessed as part of day to day clinical supervision within the department to identify when physiotherapists are ready to train in delivering telephone assessment and advice. During training the use of a ‘dummy’ system, where role playing of patients by peers or simulated patients is used and ‘live’ calls are monitored by PhysioDirect experienced staff until physiotherapists are confident and competent in using the system. Individual monitoring of clinical skills and experience was not possible for the PhysioDirect trial and so only physiotherapists with considerable experience of managing patients with musculoskeletal conditions (experienced band 6 or band 7) were invited to take part in the trial.

In the PhysioDirect trial, none of the four participating PCTs had established telephone assessment and advice services, although small-scale pilot services had been tested in two of the PCTs. As a result 21 of the 32 physiotherapists who were to deliver the new service within the randomised trial were naïve to delivering care by telephone and only three had any experience of the computer-based Huntingdon system. In preparation for the trial, the PhysioDirect team in Huntingdon developed a training and competency package which adapted the in-service PhysioDirect training that their physiotherapists receive into three distinct phases.

1. An intensive face-to-face ‘training’ session lasting for one and a half days which involved the 32 physiotherapists participating in the trial being trained by experienced PhysioDirect trainers in Huntingdon
2. A period of practice and skills consolidation using the computer-assisted system at the four PCT clinical bases, where PhysioDirect trainer support was available by telephone
3. A competency check by PhysioDirect trainers observing each physiotherapist assessing patients using the system two weeks after the intensive face-to-face training and again at six weeks if required.

The overall learning outcomes for the training are shown in Box 1.

The content of the face-to-face training session consisted of the following:

• Introduction, history and development of the PhysioDirect system
• Demonstration of the system
• Pharmacy led ‘Analgesia for Physiotherapists’
• Listening skills – exercise and role play
• Introduction of handbook and assessment forms
• Observation of experienced PhysioDirect physiotherapists taking ‘live’ calls
• Practise using a ‘dummy’ system, which utilises the clinical algorithms with mock patients
• Tips and hints for using the system effectively
• Reflection and identification of individual learning needs
• Development of work plan to address learning needs
• Plans for visits to clinical sites to establish competency

## Skill consolidation and competency checking

Establishing competency in the use of the PhysioDirect system is an integral part of the Huntingdon approach to preparing physiotherapists for working in the PhysioDirect service. The PhysioDirect software was installed at each of the PCTs in the PhysioDirect trial prior to the trial physiotherapists attending the Huntingdon training so that on return to their clinical bases the physiotherapists could practise and further develop their skills. The Huntingdon PhysioDirect trainers were available by telephone throughout this period to provide advice if problems were encountered.

A visit to each PCT was undertaken by one of the experienced Huntingdon PhysioDirect trainers approximately two weeks after completion of the training programme to see how the physiotherapists were progressing, by observing calls and facilitating a problem solving session, and to do a run through of the competency assessment, where one physiotherapist achieved competency at this early stage. A further visit was carried out approximately six weeks after the initial training, where the remaining 31 physiotherapists achieved the required competency level. The competency check consisted of the trainer assessing 50 aspects of the telephone assessment process. Each section was evaluated on a yes/no basis with overall comments about performance, issues to be addressed and an agreed action plan if required. By way of example, the competency assessment judged the physiotherapists’ ability to explore and communicate the following with callers to the service:

• Social and economic factors
• Establishment of symptoms
• Aggravating and easing factors
• Daily pattern of symptoms
• General health and special questions
• Relevant social history
• History of the current condition
• Past history
• Clinical reasoning

In addition, the manner in which the physiotherapist introduced themselves and explained the telephone assessment and advice service, and the physiotherapist's clinical reasoning and their explanation to the caller of the proposed management plan were assessed. The PhysioDirect trainers also assessed aspects of administration such as completion of the computer algorithm screens, the information sent to the patient and GP following the call, and some general facets of the telephone assessment process, such as tone of voice, use of probing questions and listening skills.

**Fig. 2:**
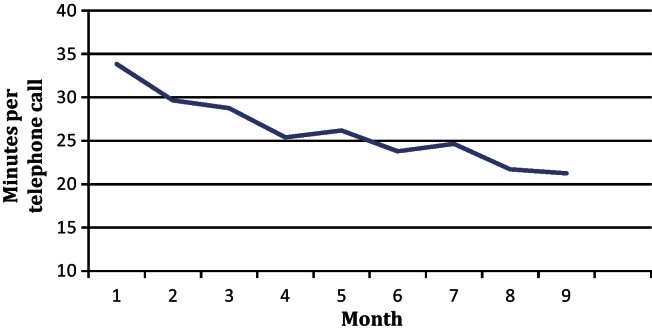
Average length of telephone calls through the course of the PhysioDirect trial.

Beyond the competency assessment, as the physiotherapists had more exposure to the PhysioDirect system, many commented how they became increasingly confident and felt more competent in assessing and advising patients over the telephone. This was reflected in the length of the telephone calls. The average call times were calculated each month from the start of a ‘run-in’ phase of the trial to the end of trial recruitment (a total of 9 months) and as shown in Fig. 2 the average length of calls reduced substantially as the physiotherapists became more proficient in using the system.

## Discussion

The aims of this paper were to describe the PhysioDirect system used in the PhysioDirect randomised trial and provide information on how physiotherapists were prepared for using the system and delivering the service, including the training and competency assessment process.

The observations made in preparing staff, over many years, to deliver a telephone assessment and advice service in Huntingdon have been reinforced by the understanding gained from training a group of physiotherapists for a pragmatic randomised trial who were largely naïve to the delivery of telephone based services. Even for experienced musculoskeletal physiotherapists, adequate support to deliver telephone assessment and advice services was clearly essential. This included training in the new system, enhancing skills such as listening and communication, opportunities to consolidate new skills through practice and demonstrating competency in the new approach.

Training in the use of a telephone assessment and advice system was especially important as it utilised an unfamiliar medium for recording clinical information i.e. an electronic rather than a paper based system. However, an essential element of preparing staff for delivering telephone assessment and advice services was the opportunity to consolidate new skills with a period of practice using peers or simulated patients. Physiotherapists involved in both the trial and the Huntingdon service demonstrated the benefit of a period of practice before feeling confident and competent to deliver care in this way. Models of skill acquisition describe progression through several stages from ‘novice’ to ‘expert’ in practical skills such as car driving or playing chess [7] but these have also been applied to the acquisition of clinical skills by health care practitioners [8,9]. Of note is that expertise takes time to develop and emerges through repeated experience rather than being something that can be ‘taught’ in formal education, and that the skills acquired are context specific and do not necessarily translate from one situation to another [10]. This model is supported by the experiences from the current study and Huntingdon service where even physiotherapists with considerable experience in managing patients with musculoskeletal problems benefited from a period of skill consolidation.

The training and skill consolidation process also served to allay reservations about delivering care by telephone, where physiotherapists cite concerns about patient safety and their fear of missing an important diagnosis [1], despite evidence that physiotherapists reach the same diagnoses whether using telephone or face-to-face assessment [11]. By discussing concerns with their peers and by directly observing experienced PhysioDirect physiotherapists in their assessment and management of patients using the telephone physiotherapists were effectively reassured.

## Benefits of the computerised system

In the context of the PhysioDirect trial, the computerised system facilitated standardisation of the intervention, by utilising a set number of algorithms and management pathways. Although telephone assessment and advice services can be offered using traditional paper based record keeping the computerised system does have several advantages which include:

• Providing real time prompts for special questions pertinent to the clinical condition being assessed
• Highlighting appropriate management pathways when the diagnosis has been made
• Providing efficient data management as stored data can be accessed easily from any location, which is particularly beneficial if the service is not based in only one location, or if previous paper case records may be stored elsewhere, and are not easily retrievable when a patient telephones for further advice or for a new problem
• Facilitating audit of the service as the system can provide reports on various aspects of the service

## What are the implications for new PhysioDirect services?

When setting up a physiotherapy-led telephone assessment and advice service, it is clear that time and resources for training of staff and skill consolidation need to be factored into the implementation plan. Allowing staff to become familiar with the system and feeling confident in delivering care by telephone will reduce concerns or anxiety in those providing the service. An appropriate ‘run-in’ period contributes to more efficient delivery of the service, which was highlighted by several of the trial physiotherapists commenting on the challenge of listening, typing and clinically reasoning at the same time, when they started using the PhysioDirect computer system. This was reflected in the steady reduction in time spent on each telephone consultation as the trial progressed from an average of 34 to 22 minutes. In addition, as staff move on to different posts, and until telephone services are commonplace, it is also important that the training and support system is sustainable within the service and available to new staff.

Over years of training staff to work in PhysioDirect telephone assessment and advice services, the team at Huntingdon have noted that some individuals find adapting to delivering telephone-based services more challenging than others. In general the Huntingdon experience suggests that a high level of clinical experience is required before staff can deliver such telephone based services safely and effectively and in the context of the PhysioDirect trial only band 6 and band 7 physiotherapists with musculoskeletal experience were invited to take part. There is some evidence that clinical experience influences the management decisions of physiotherapists during telephone compared to face to face consultations, with decisions of inexperienced therapists being less in agreement with face to face decisions compared to those of experienced staff [10]. The computerised system does not replace clinical experience, as although the system prompts questions about each condition and manages the assessment information in a convenient and auditable format, the physiotherapist is still required to process the information and use their clinical reasoning skills and knowledge to reach a diagnosis and provide appropriate information to the patient on management of the presenting condition.

A further consideration for allocating staff is that most physiotherapists are reluctant to spend a majority of their clinical time engaged in telephone assessment and advice. It is therefore recommended that only a percentage of patient contact time of each physiotherapist is spent engaged in telephone delivered care [12,13]. In the PhysioDirect trial, no physiotherapist spent more than 50% of their working week offering the new service.

## Conclusion

When setting-up or providing physiotherapy-led telephone assessment and advice services for musculoskeletal patients, training and support for physiotherapists delivering care in this way is essential. An enhanced skill set is required for telephone assessment and advice particularly in listening and communication skills, which also enhances traditional face-to-face consultations. In addition to learning the structure of the new computer-based system, physiotherapists should have the opportunity to consolidate their skills to become proficient and confident in assessing patients and delivering care using the telephone. A computer-based system assists the delivery of telephone based physiotherapy advice and treatment.

## Figures and Tables

**Table 1:** Patient categories and subsequent management pathways resulting from PhysioDirect assessment.

Patient category	Management pathway
Specific musculoskeletal problem and the assessment indicates the patient can be given a sound explanation of the musculoskeletal problem	Patient is given clear verbal explanation and advice. Information sent in the post to facilitate self-management.
a. If the problem is highly likely to resolve	a. Instructed to call back if the condition does not resolve
b. The self-management advice may well help but a follow-up appointment may be required	b. Patient is instructed to call back after a specified length of time
The presenting problem is musculoskeletal but there are indications of serious pathology present	Specific pathway followed for presentation, e.g. cauda equina
Specific musculoskeletal condition but face-to-face assessment required e.g. neurological status assessment for patients with radiculopathy	Patient booked into clinic for face-to-face appointment for further examination and assessment
Not possible to make a sound diagnosis with telephone assessment	Patient booked for face-to-face appointment to clarify the diagnosis and commence appropriate management
The primary presenting problem is not musculoskeletal	Patient referred back to general practitioner

## Box 1 The learning outcomes for the training package

Following training and practice, physiotherapists will be able to;

• Demonstrate enhanced listening and interview skills to assess and manage patients safely and appropriately using the PhysioDirect computerised system
• Identify and signpost patients with serious pathology which is outside the scope of management by physiotherapists to the most appropriate service or back to the caller's GP
• Use structured clinical reasoning to make an appropriate clinical diagnosis
• Demonstrate excellent verbal skills to discuss, explain and agree a management plan for callers to the service
• Utilise the medication algorithm to provide appropriate advice on over the counter medication or discuss appropriate medication with the caller's GP
• Reach an appropriate decision to bring a patient in for face-to-face assessment where a clinical diagnosis cannot be made over the phone or where further examination is required, for example to assess neurological status
